# Etymologia*:* Knemidocoptic Mange

**DOI:** 10.3201/eid2010.ET2010

**Published:** 2014-10

**Authors:** Begaleaon Helene Somda

**Keywords:** etymologia, knemidocoptic mange, mange, mites, knemidocoptiasis, birds

## Knemidocoptic mange [neʺmĭ-do-kopʹtik mānj]

From the Latin *manducare* (to itch), mange is a skin disease caused by mites in domestic and wild animals. Knemidocoptic, from the Greek *knemid* (greave, a piece of armor that protects the leg) and *koptein* (to cut), refers to the morphology and pathogenesis of mites of the genus *Knemidocoptes*, which are burrowing mites of birds. Commonly known as scaly face, scaly legs, or tassel foot, knemidocoptiasis affects primarily the face and legs of birds around the world worldwide and can be fatal.
